# An exploration of the protective effect of rodent species richness on the geographical expansion of Lassa fever in West Africa

**DOI:** 10.1371/journal.pntd.0009108

**Published:** 2021-02-01

**Authors:** Kyung-Duk Min, Jusun Hwang, Maria Cristina Schneider, Yeonghwa So, Ju-Yeun Lee, Sung-il Cho

**Affiliations:** 1 Institute of Health and Environment, Graduate School of Public Health, Seoul National University, Seoul, Republic of Korea; 2 Wildlife Conservation Society, Bronx, New York, United States of America; 3 Department of International Health, School of Nursing and Health Sciences, Georgetown University, Washington DC, United States of America; 4 Institute of Collective Health Studies, Federal University of Rio De Janeiro, Rio De Janeiro, Brazil; 5 Department of Public Health Science, Graduate School of Public Health, Seoul National University, Seoul, Republic of Korea; University of Surrey, UNITED KINGDOM

## Abstract

**Background:**

Lassa fever (LF) is one of the most devastating rodent-borne diseases in West Africa, causing thousands of deaths annually. The geographical expansion of LF is also a concern; cases were recently identified in Ghana and Benin. Previous ecological studies have suggested that high natural-host biodiversity reduces the likelihood of spillover transmission of rodent-borne diseases, by suppressing the activities of reservoir species. However, the association of biodiversity with the geographical expansion of LF has not been the subject of epidemiological studies.

**Methodology/Principal findings:**

We conducted a spatial analysis based on sociodemographic, geographical, and ecological data, and found that higher rodent species richness was significantly associated with a lower risk of LF emergence in West Africa from 2008 to 2017 (Odds Ratio = 0.852, 95% Credible Interval = 0.745–0.971).

**Conclusions/Significance:**

The results reinforce the importance of the ‘One Health’ approach by demonstrating that a high level of biodiversity could benefit human health.

## Author summary

Rodent diversity has been studied as a protective factor for rodent-borne diseases and rodent-related tick-borne diseases, such as hantavirus infection and Lyme diseases, in human population. The protective effect is called dilution effect and based on the ecological mechanism that the high diversity of rodent species could suppress the activities of reservoir rodents thereby reduce the likelihood of spillover transmission from rodents. However, the effect has been studied mostly for endemic diseases, and the effect on the geographical expansion of rodent-borne diseases has not been the subject of epidemiological studies. Considering that the dilution effect may also be applicable to the geographic expansion of rodent-borne infectious diseases in that a high rodent species diversity could decrease migration of infected rodents from the disease-endemic area, we examined the association between rodent species richness and the geographical expansion of Lassa fever (LF) in West African countries. Analyzing the regions without LF until 2007, the results showed that the region with lower number of rodent species showed higher risk of emerging LF cases from 2008 to 2017, implying the protective effect of higher rodent species richness on spatial expansion of LF. The results reinforce the importance of the ‘One Health’ approach by demonstrating that a high level of biodiversity could benefit human health.

## Introduction

Lassa fever (LF) is an acute viral hemorrhagic fever transmitted by *Mastomys natalensis*, a multi-mammate rat, which is broadly distributed across sub-Saharan Africa. The annual number of cases has been estimated based on a longitudinal study as 100,000–300,000, with 5,000 deaths, although there is a large uncertainty with these estimates. [1,2]. Recently, the number of LF cases surged in Nigeria [3], and the unprecedented size of the outbreak caused severe social and economic dislocation at the national level. Moreover, LF have also caused a global concern, as Germany [4], the United Kingdom [5] and the United States [6] reported imported LF cases. In Germany, secondary local infection from an imported case also reported [7]. Because of its relatively long incubation period (6–21 days [8]) and the global increase in the use of air transportation, LF importation represents a considerable threat to public health, globally.

Interestingly, the distribution of LF is limited to West Africa; there has been no report of autochthonous LF in sub-Saharan Africa, where the reservoir species are distributed. Lassa fever virus (LASV) originated 1,000 years ago in Nigeria, and only recently spread to the western region known as the Mano River Union (MRU), which encompasses Cote d’Ivoire, Guinea, Liberia, and Sierra Leone [9]. However, there have been relatively few LF cases between Nigeria and the MRU, implying the existence of factors that limit the distribution of LF to certain regions.

Geographical barriers may explain the limited distribution of LF. Siddle *et al*. [10] detected genetic variations of LASV among regions partitioned by rivers in Nigeria, and suggested that such geographical barriers suppress migration of reservoir rodents. The mountain chain between Nigeria and Cameroon could also explain the lack of reports of autochthonous LF cases in Cameroon during a large-scale outbreak in Nigeria. However, these factors cannot account for the absence of LF in other parts of West Africa; *i*.*e*., the countries between MRU and Nigeria. A recent study [11] proposed a co-evolution hypothesis as an alternative explanation. The hypothesis states that the genetic variation of reservoir rodents in West Africa could result in differences in the ability to transmit the virus. Redding *et al* [12] suggested that only one clade of *M*. *natalensis* (Western clade) can host LASV by phylogeographic evidence. However, the hypothesis has not been confirmed given the broad host range of LASV [13], such as *Hylomyscus pamfi* and *Mastomys erythroleucus* [14].

Other factors can be associated with the geospatial distribution of LF in West Africa. For example, climate factors, especially for precipitation or humidity, can affect the distribution of LF. Fichet-Calvet and Rogers [15] found that emergence of LF was associated with humidity possibly because LASV survival is better in humid environment and also rodent becomes active in a rainy season. Deforestation and anthropogenic environmental disruption could also increase contact between reservoirs and human, which subsequently lead to increase LF cases [16]. In addition, level of surveillance sensitivity can affect the distribution of reported LF. While weak surveillance system for LF is generally found in many West African countries [17], recent implementation of Regional Disease Surveillance Systems Enhancement Project which aimed to strengthen infectious disease surveillance capacity in West Africa [18] could increase LF reports.

Rodent species richness; *i*.*e*., the number of rodent species, may explain the geographic limitation of LF. A high rodent species diversity could suppress the abundance and activities of each rodent species [19], including *Mastomys natalensis*, thus reducing both the disease prevalence among rodents and its transmission to humans; this is known as the dilution effect [19]. There are controversies on the generality of the dilution effect. For example, one of necessary conditions for dilution effect is that suitability of high-competency species in low diversity environment should be better than other host species. In the regions where the underlying condition is not satisfied, low diversity could even decrease spillover transmission risk [20]. However, in case of LF in West Africa, the necessary condition is highly likely to be satisfied because *M*. *natalensis* showed the strongest competency among local rodent species and resilient in modified environment [21]. The dilution effect has been discussed mostly for endemic diseases [22,23], but it may also be applicable to the geographic expansion of rodent-borne infectious diseases in that a high rodent species diversity could decrease migration of infected rodents from the disease-endemic area. In this study, we examined the association between rodent species richness and the geographical expansion of LF in West African countries based on spatial data from multiple sources.

## Methods

### Study design and the study unit

**Fig 1:**
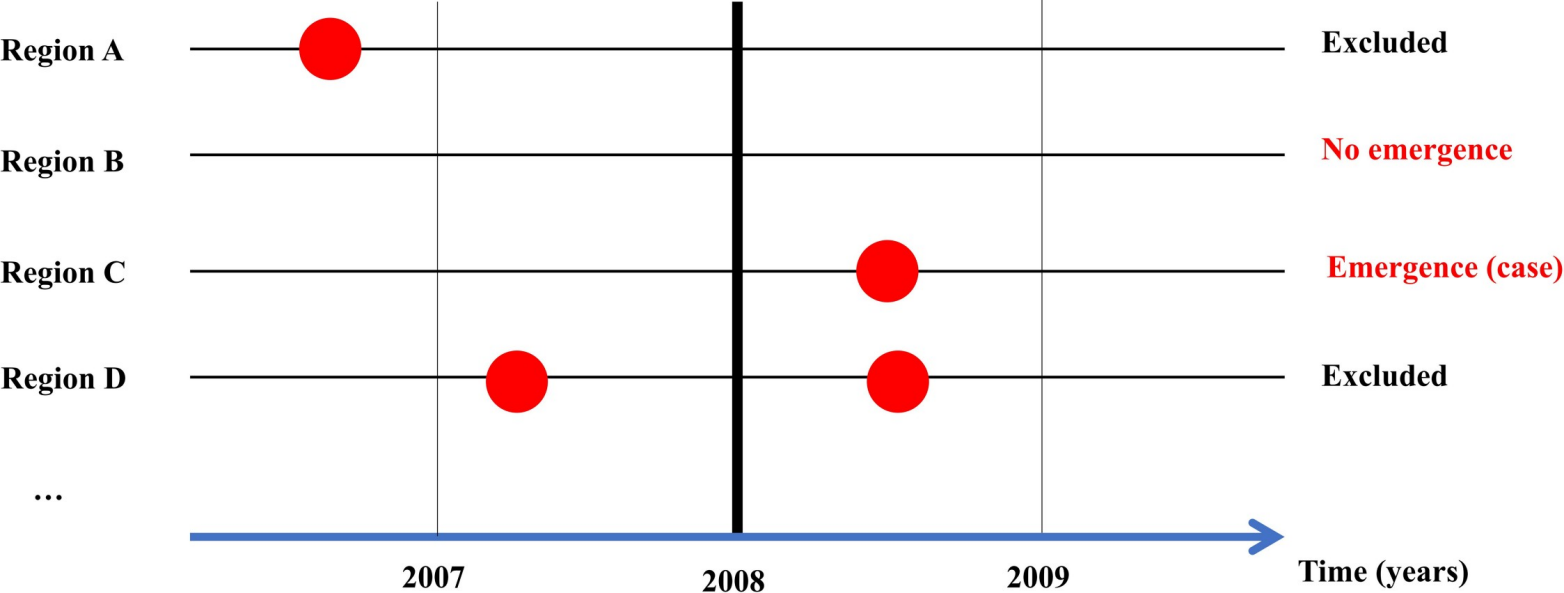
Study design. The outcome of interest was Lassa fever (LF) emergence events over a 10-year period beginning in 2008, and the main explanatory variable was rodent species richness. Regions with LF cases reported before 2008 were excluded from the analysis; we compared rodent species richness between regions with LF cases from 2008 to 2017 and those with no reported LF cases up to 2017. Regions A and D had LF cases before 2008, so were excluded from the analysis. In Region C, LF emerged after 2008. We compared rodent species richness between Regions C and B.

We examined the association of rodent species richness with LF emergence events. Although there are several criteria for defining an emerging infectious disease (*e*.*g*., a novel pathogen, mutation, or drastic increase in incidence), the spread of LF to regions with no reported human cases was used herein. Specifically, cases emerging after 2008 were analyzed, because the geographical distributions of rodent species in the West Africa countries were assessed after 2008 (Table A in S1 Text), and distribution data are needed to calculate rodent species richness. This study was conducted over the 10-year period of 2008 to 2017; LF emergence events during this period was an outcome variable. Accordingly, historical LF cases prior to 2007, and those from 2008 to 2017, were extracted from the data. We compared the rodent species richness between regions with and those without LF emergence events, excluding the regions with LF cases reported before 2008 (Fig 1).

**Fig 2:**
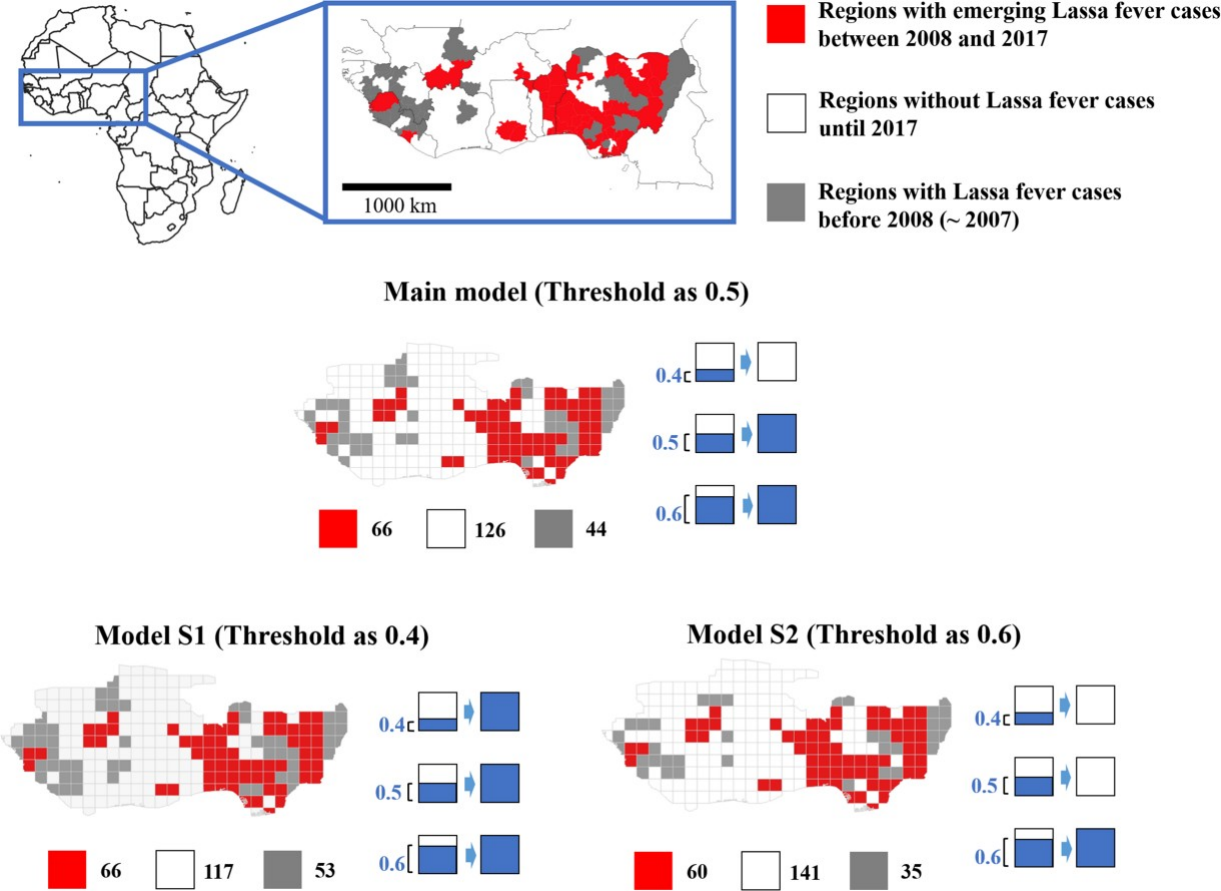
Study area and study units. Based on web-based surveillance data and a prior review, historical Lassa fever (LF) cases (up to 2017) were analyzed. Provinces in West Africa were categorized as follows: grey, outbreak reported before 2007; red, LF emergence events over a 10-year period beginning in 2008; and white, no human LF cases up to 2017. We created a 1 × 1° grid map, and categorized the grids based on their intersection with the provinces. For example, using a 50% threshold, if a grid consisted of 60% province A and 40% province B, it was considered to be province A. We created two datasets using thresholds of 40% and 60% to confirm the robustness of the results. Made with Natural Earth.

The study area was defined based on two criteria. First, we selected regions suggested to harbor the main reservoir species, *Mastomys natalensis*, because cases of autochthonous LF are unlikely in regions without the reservoir. Second, we selected only West African countries with at least one autochthonous LF case up to 2017, under the assumption of a surveillance capacity sufficient for detecting and reporting LF (Fig 2 and Fig A in S2 Text).

### Data acquisition and preprocessing

We acquired data on historical LF cases, rodent species richness, and sociodemographic, climate, land cover, and geographic factors from various sources (Table 1). Historical LF cases were obtained from a review [13] and the World Health Organization Disease Outbreak News (WHO DON) [24]. These data were supplemented with other informal web-based surveillance data (ProMed-Mail [25] and Health map [26,27]) because of the insufficient surveillance capacity associated with the resource-limited setting of this study. Although these are not national-level surveillance data, prior epidemiological [28,29] and infectious-disease detection [30] studies also used a multiple-source approach. Details of the web-based surveillance data and historical LF events are provided in S3 Text. We listed the historical LF cases by year and province. The provinces in the study area were categorized into regions with LF cases before 2007, regions with LF emergence events from 2008 to 2017, and regions with no LF cases before 2017. Next, we created 1 × 1° grid maps, and categorized the grids based on their intersection with the province map. Using a 50% threshold, if a grid consisted of 60% province A and 40% province B, for example, it was considered to be province A (Fig 2).

**Table 1:** Data sources used in this study.

Variable	Data source	Ref(s)
**Lassa fever outbreak**	Gibb *et al*, ProMed-MailHealthmap, WHO DON	[13,24–27]
**Rodents species richness**	IUCN	[31]
**Mammalian predator species richness**	IUCN	[31]
**Avian predator species richness**	Birdlife	[32]
**Human footprint score**	Venter O et al	[33]
**Forest cover**	GFC	[34]
**Agricultural land use**	Tuanmu et al	[35]
**Elevation**	SRTM	[36]
**Precipitation**	Worldclim	[37]
**Temperature**	Worldclim	[37]
**Population**	Worldpop	[38,39]
**GDP** ^**a**^	Kummu et al	[40]
**Total space** ^**b**^	-	

^a^ GDP, gross domestic product

^b^ Total space was calculated directly from the polygon map.

The International Union for the Conservation of Nature (IUCN) [31] has developed a comprehensive spatial database of species’ ranges, including terrestrial mammals. The geographical range of avian species was obtained from Birdlife [32]. The mammalian and avian species range data are in polygon format. To calculate the species richness, we intersected polygons and counted the number of species whose distribution range occupied more than 50% of a given grid. *Rodentia* species were included in the calculation of rodent species richness, and *Carnivora*, *Accipitridae*, *Falconidae*, and *Strigidae* species in that of predator species richness.

As sociodemographic factors, population density [38,39] and gross domestic product (GDP) [40] were analyzed and adjusted for. Population density data were acquired for 2010 and 2015 and GDP data for 2012 and 2013, and averaged as representative values for the study period. The human footprint score [33] was used to assess anthropogenic pressure on the environment. The score ranged from 0 to 50, and a higher score indicated greater anthropogenic pressure. We used the score for 2009, because only data for 1997 and 2009 were provided. These data are in raster format. We extracted the value of each raster cell, and used it to calculate the population density, GDP per capita, and human footprint score in a given grid area.

To adjust for potential confounding effects, global climate data from 1970 to 2000 were obtained from WorldClim v. 2.0 [37], which provides monthly average temperature and precipitation data at a resolution of 1 km per pixel in raster format. The annual average temperature and annual precipitation were calculated for each grid area.

In terms of geographic variables, forest cover, agricultural land use, and elevation land cover data were acquired from Tuanmu *et al*. [35]. They analyzed four global land cover products: DISCover, GLC2000, MODIS2005, and GlobCover. Agricultural land use data were obtained in raster format. The range of each raster cell was 0 to 100, representing the probability of land cover. We used a threshold value of 50 (*i*.*e*., a 50% probability of agricultural land use) to assess agricultural land use in a given raster cell. In terms of forest land cover, raster data from Global Forest Change (GFC) [34] were used. The forest cover variable ranged from 0 to 100 and was in raster format; the value represents the probability of a tree canopy. As in a previous study [41], we used 50 as the threshold for the presence of forest in a given raster cell. We summed all raster cell values in each grid area to estimate the area of each type of land use in a given grid area. Elevation data were obtained from Shuttle Radar Topography Mission (SRTM; v. 4.1) [36], which provides 90-meter-scale global elevation data in raster format. We averaged the values for each grid.

R software (v. 3.5.1) [42] was used for preprocessing. The “rgdal” package [43] was used for shape file importation and the “raster” package [44] was employed to intersect polygons and extract raster values. Parallel computing was performed using the “doParallel” package [45]. All dataset used in this study can be found in S1 Data.

### Statistical analysis

We evaluated the general characteristics of grids with and without LF emergence events. The mean and standard deviation of each variable were calculated, and choropleth maps with decile values were generated. To avoid multicollinearity, variables with a variance inflation factor (VIF) value of > 10 [46] and a one-to-one correlation coefficient of > 0.8 were excluded from the analysis.

Logistic regression models were used to examine the association between LF emergence events and rodent species richness. A generalized linear model (GLM) with spatial autocorrelation was employed. The equation can be expressed as below: $\ln\left(\frac{p}{1-p}\right)=\alpha +\beta_{i}X_{i}+v_{i}+\varepsilon_{i}$ where p is a probability of LF emergence; α is the intercept; βi is the regression coefficient; Xi is the set of explanatory variables; υi is the structured spatial random effect for grid i; εi is the non-spatial random effect for grid i. A Bayesian approach with integrated nested Laplace approximation [47] was applied using the “R-INLA” package [48]. The results of the fully adjusted models are shown as odds ratios (ORs) and 95% credible intervals (95% CIs). Area under the curve (AUC) values were estimated based on the mean value fitted to the model. To evaluate uncertainty caused by mismatch between the grid and province maps, we conducted a sensitivity analysis using thresholds of 40% and 60% to assess the robustness of the results.

Considering that OR can seriously exaggerate Relative risk (RR) unless rare disease assumption is satisfied, we planned to conduct Zou’s modified Poisson regression [49] if the proportion of grid with emergence cases is small. Although Poisson regression model can estimate RR directly, the error for the estimated RR can be exaggerated in binomial data analysis. Zou proposed a modified Poisson regression method to deal with this issue by using a robust error variance procedure.

## Results

### Descriptive analysis

The general characteristics of the grids are shown in Table 2. The rodent species richness was lower in the grids with versus without LF emergence events (12.79 and 14.73, respectively). Also, the population density and GDP per capita were higher in the regions with LF emergence events (158.74 per km^2^ and 3,833.37 USD per capita, respectively) than in those without LF emergence events (56.79 per km^2^ and 2,359.56 USD per capita, respectively). In terms of climate, the regions with LF emergence events had higher annual precipitation (1,282.79 mm) than did those without LF emergence events (1,086.44 mm), but the temperature was higher in non-emergence than in emergence regions (27.18°C and 26.66°C, respectively). The proportion of forest cover was higher in the non-emergence regions (10.81%) than in emergence regions (7.64%); the proportion of agricultural land use also differed (43.13% in emergence regions, 36.35% in non-emergence regions). The spatial distributions of the variables are shown in Fig A-J in S4 Text.

**Table 2:** Descriptive analysis results.

Variables	With LF ^a^ (N = 66)	Without LF ^a^ (N = 126)
	Mean (±SD)	
Rodent species richness	12.79 ± 3.5	14.73 ± 3.7
Predator species richness	36.52 ± 3.8	34.35 ± 6.6
Human footprint score ^b^	10.8 ± 3.1	8.57 ± 2.9
Forest cover (%)	7.64 ± 15.3	10.81 ± 24.0
Agricultural land use (%)	43.13 ± 34.2	36.35 ± 33.2
Elevation (m)	292.78 ± 153.8	271.98 ± 110.0
Precipitation (mm)	1282.79 ± 556.6	1086.44 ± 605.8
Temperature (°C)	26.66 ± 0.8	27.18 ± 1.2
Population density (per km^2^)	158.74 ± 228.0	56.79 ± 87.7
GDP ^c^ per capita (USD)	3833.37 ± 1982.9	2359.56 ± 1353.1
Total space (1000 km^2^)	14.17 ± 1.1	14.12 ± 1.0

^a^ LF, Case report of Lassa fever during the study period (2008–2017).

^b^ The human footprint score ranged from 0 to 50; a higher score indicates greater anthropogenic pressure on the environment.

^c^ GDP, gross domestic product

### Assessment of multicollinearity

The VIF values were calculated to assess multicollinearity. None of the variables had a VIF value of > 10, so none were excluded. In the one-to-one correlation analysis, all combinations of variables had a low or moderate correlation, and none were excluded (Table A and Fig A in S5 Text)

### Association between rodent species richness and LF emergence

The rodent species richness showed a significant negative association with LF emergence events (OR = 0.852, 95% CI 0.745–0.971), but its association with predator species richness (OR = 1.090, 95% CI 0.972–1.226) was not significant (Table 3). In a sensitivity analysis using different thresholds to generate grid maps (0.4 and 0.6 for models S1 and S2, respectively), negative associations were also found (OR = 0.864, 95% CI 0.745–0.987 and OR = 0.870, 95% CI 0.766–0.988 for models S1 and S2, respectively). The AUC for all models was > 0.81, indicating an excellent fit to the data.

**Table 3:** Association between rodent species richness and LF emergence events.

Variable	Odds ratio (95% Credible Interval)		
	Main model	Model S1	Model S2
Rodent SR ^a^	0.852 (0.745–0.971)	0.864 (0.754–0.987)	0.870 (0.766–0.988)
Predator SR^a^	1.090 (0.972–1.226)	1.092 (0.969–1.233)	1.073 (0.965–1.195)
Human foot print	1.141 (0.853–1.527)	1.173 (0.876–1.571)	1.106 (0.841–1.456)
Forest cover	0.541 (0.016–16.239)	0.307 (0.007–12.429)	0.437 (0.014–12.111)
Agricultural land use	0.684 (0.078–5.736)	0.858 (0.099–7.185)	1.084 (0.136–8.348)
Elevation	1.002 (0.997–1.007)	1.003 (0.997–1.008)	1.000 (0.995–1.005)
Precipitation	1.001 (1.000–1.002)	1.001 (1.000–1.003)	1.001 (0.999–1.002)
Temperature	0.887 (0.414–1.890)	0.769 (0.353–1.662)	0.792 (0.376–1.654)
Population density	1.006 (0.999–1.013)	1.005 (0.998–1.011)	1.004 (0.999–1.009)
GDP ^b^ per capita	1.000 (1.000–1.001)	1.000 (1.000–1.001)	1.000 (1.000–1.001)
Total space	1.001 (1.000–1.002)	1.001 (1.000–1.002)	1.000 (1.000–1.001)
AUC ^c^	0.841	0.849	0.810

^a^ SR, Species richness

^b^ GDP, gross domestic product

^c^AUC, area under the curve

*Note*: The analyses were conducted using multivariable logistic regression models with consideration of spatial autocorrelation. The outcome variable was LF emergence events. The grid size was 1 × 1°. The number of grids with and without LF emergence was 66 (cases) and 126 (controls), respectively; the total number of grids was 192. The rodent and predator variables indicate species richness.

The OR we estimated can exaggerate the relative risk (RR) because the model included a large number of cases (the OR is similar to the RR only if the number of cases is small, according to rare disease assumption). To address this issue, the modified Poisson regression proposed by Zou [49] was used and the RR was estimated as 0.915 (95% CI = 0.856–0.978). Because the interquartile range (IQR) of the rodent species was 5 (first quartile, 12; third quartile, 17), the RR for IQR decrease in richness was 1.56 (= (1/0.915)^5^, 95% CI = 1.11–2.18), representing a 56% higher risk of emergence in the regions where IQR lower rodent richness is shown.

## Discussion

We examined the association between rodent species richness and emerging LF events, defined as geographic expansion in West Africa over a 10-year period beginning in 2008. The hypothesis was that a higher rodent species richness suppresses the activities of reservoir rodents, and thus reduces the migration of LF-infected reservoirs from an endemic area. The results showed that a higher rodent species richness was negatively associated with LF emergence, and the association was robust irrespective of the method used to categorize the regions.

The negative association between rodent species richness and the risk of LF emergence suggested that the dilution effect [23] can apply to the spatial spread of infectious diseases. The high richness environment could reduce activities of reservoir rodents, and consequently reduce LF prevalence among reservoir species. Considering that incidence of human LF was associated with the prevalence in rodents [50], The low possibility of human LF case report in high rodent richness area can be mediated by low prevalence among rodents. However, the association reported herein may not generalize to other rodent-borne diseases, as not all pathogens show high host specificity as LASV. Although other species, such as *Hylomyscus pamfi* and *Mastomys erythroleucus* [14], have been proposed as the natural host of LASV, LASV has greater host specificity than other rodent-borne diseases, such as hantaviruses.

The predator species richness did not show a significant association with the risk of LF emergence. This is not consistent with prior ecology [51] and disease ecology [22,52] studies on the regulatory effect of predator species richness on rodent activities. The inconsistency may be explained by composition of rodent prey species of predators in these regions. The primary reservoir of LF, *M*. *natalensis*, is not a dominant rodent species in West Africa [53]. Considering that predation pressure tends to focus on dominant species [54], the effect of predator richness is unlikely to have led to noticeable differences in rodent activity. As an exploratory ecological study, there are several limitations that should be noted. First, we did not consider the temporal changes of species richness from 2008 to 2017. However, the temporal fluctuation in rodent species richness is unlikely to be considerable, as most rodent species we included showed “LC” class, meaning low probability of extinction. Second, some important species were not analyzed. For example, several insectivore species are in the same trophic hierarchy as rodents, with which they compete, thus limiting the rodent population size. Third, we did not include relative abundance of individual rodent species as a measurement for rodent diversity and only counted the number of species. Although investigating relative abundance of individual rodent species in whole West African region could not be practical at this moment, additional studies with different diversity measures (e.g. Simpson diversity index) are worthwhile to be conducted. Fourth, considering that we did not consider the abundance of reservoirs in this study, we cannot exclude the possibility that the absence of LF in many West African countries could be derived from low abundance of reservoirs. However, various factors that are considered to be associated with reservoir abundance, such as level of human modification [21], vegetation index, temperature and elevation [55] were adjusted in our models. In addition, Olayemi *et al* [50] found that reservoir abundance could be high in some non LF endemic area implying that the abundance of reservoir would have limited effect on the LF emergence. Fifth, a recent LF risk map from Mylne *et al* [55] suggested that presence of LF is possible in Senegal and Niger where we excluded in the models. Inclusion of those countries in the model could change our results, although a recent review study [56] did not find the evidences of LF presence in those countries. Sixth, we considered *M*. *natalensis* as a major reservoir in this study, but other possible reservoirs such as *Rattus rattus* and *Mus musculus* can be included in the follow-up studies [57]. Seventh, our study design was based on macroscopic scale as the study unit was 1 × 1° grid. Future studies with microscopic scale can supplement our results. Marien *et al* [58] found that there is spatial heterogeneity in LF prevalence among rodents on a household level. Considering that high LF prevalence among rodents is associated with higher probability of spillover transmission, microscale heterogeneity within our study unit is highly expected [57]. Eighth, we did not include outbreak information from individual countries’ health authorities. However, considering surveillance capacity may vary in different countries, our data collection process using multiple sources including ProMed-Mail, Healthmap, and WHO DON can minimize potential underreporting bias. Lastly, we used 1 × 1° grid squares; use of a different scale (*e*.*g*., 0.5 × 0.5° grid squares) would have resulted in a different sample size, thus affecting the values of explanatory variables. This is known as the modifiable area unit problem.

The results have important implications for public health and environmental conservation. From a public health perspective, the results could be used to predict areas at risk of LF emergence in West Africa. Although prediction of the risk from the results of current study alone cannot be reliable, further studies that incorporate other predictors such as population explosion, anthropogenic invasion on nature can improve the outcome. In addition, the results can be used for advocating ecosystem services, as rodent diversity could be dependent on conservation efforts at the local level. Considering the limited effect of anthropogenic rodent control on reducing LF incidence [59], holistic approach incorporating environmental aspect can be essential to minimize the impact from LF.

## Supporting information

S1 Data All dataset used in this study. (CSV)

S1 Text **Table A.** Rodent species included. **Table B.** Mammalian predator species included. **Table C.** Avian predator species included.
(DOCX)

S2 Text **Fig A**. **Selection process for study area.** Note: Made with Natural Earth.
(DOCX)

S3 Text **Table A**. The list of Lassa fever outbreak regions.
(DOCX)

S4 Text **Fig A. Distribution of rodent species richness.** *Note*: The grids (n = 192) were categorized into quintiles of rodent species richness to visualize the geographic distribution. Grey grids (n = 44) indicate regions with incident LF before 2008, which were excluded from the analysis. Made with Natural Earth. **Fig B. Distribution of predator species richness.** *Note*: The grids (n = 192) were categorized into quintiles of predator species richness to visualize the geographic distribution. Grey grids (n = 44) indicate regions with incident LF before 2008, which were excluded from the analysis. Made with Natural Earth. **Fig C. Distribution of human footprint score**. *Note*: The grids (n = 192) were categorized into quintiles of human footprint score to visualize the geographic distribution. Grey grids (n = 44) indicate regions with incident LF before 2008, which were excluded from the analysis. Made with Natural Earth. **Fig D. Distribution of proportion of forest land cover.** *Note*: The grids (n = 192) were categorized into quintiles of proportion of forest land use to visualize the geographic distribution. Grey grids (n = 44) indicate regions with incident LF before 2008, which were excluded from the analysis. Made with Natural Earth. **Fig E. Distribution of proportion of agriculture land use**. *Note*: The grids (n = 192) were categorized into quintiles of proportion of agricultural land use to visualize the geographic distribution. Grey grids (n = 44) indicate regions with incident LF before 2008, which were excluded from the analysis. Made with Natural Earth. **Fig F. Distribution of elevation**. *Note*: The grids (n = 192) were categorized into quintiles of elevation to visualize the geographic distribution. Grey grids (n = 44) indicate regions with incident LF before 2008, which were excluded from the analysis. Made with Natural Earth. **Fig G. Distribution of annual precipitation.** *Note*: The grids (n = 192) were categorized into quintiles of annual precipitation to visualize the geographic distribution. Grey grids (n = 44) indicate regions with incident LF before 2008, which were excluded from the analysis. Made with Natural Earth. **Fig H. Distribution of annual mean temperature.** *Note*: The grids (n = 192) were categorized into quintiles of annual mean temperature to visualize the geographic distribution. Grey grids (n = 44) indicate regions with incident LF before 2008, which were excluded from the analysis. Made with Natural Earth. **Fig I. Distribution of population density.** *Note*: The grids (n = 192) were categorized into quintiles of population density to visualize the geographic distribution. Grey grids (n = 44) indicate regions with incident LF before 2008, which were excluded from the analysis. Made with Natural Earth. **Fig J. Distribution of GDP per capita.** *Note*: The grids (n = 192) were categorized into quintiles of GDP per capita to visualize the geographic distribution. Grey grids (n = 44) indicate regions with incident LF before 2008, which were excluded from the analysis. Made with Natural Earth.
(DOCX)

S5 Text **Table A.** Variance inflation factor (VIF) values of the explanatory variables. **Fig A. One-to-one correlations of selected variables.** *Note*: A, rodent species richness; B, predator species richness; C, human footprint score; D, proportion of forest land use; E, proportion of agricultural land use; F, elevation; G, total space; H, annual precipitation; I, annual mean temperature; J, population density; K, gross domestic product (GDP) per capita.
(DOCX)
