# Supplementary material for: An exploration of the protective effect of rodent species richness on the geographical expansion of Lassa fever in West Africa
Source: PLoS Negl Trop Dis. 2021 Feb 1;15(2):e0009108. doi: 10.1371/journal.pntd.0009108 (PMC7877741; doi:10.1371/journal.pntd.0009108)
Supplement: S1 Text — Table A. Rodent species included. Table B. Mammalian predator species included. Table C. Avian predator species included. (DOCX) [file pntd.0009108.s002.docx]

**S1 Appendix. Wildlife species included in this study**

The rodent, mammalian predator, and avian predator species included in the calculation of species richness are listed in Tables A, B, and C, respectively.

**Table A. Rodent species included**

| **Academic names** | **Year of assessment** | **IUCN** |
| --- | --- | --- |
| *Mastomys natalensis* | 2008 | LC |
| *Mus haussa* | 2016 | LC |
| *Xerus erythropus* | 2008 | LC |
| *Arvicanthis niloticus* | 2008 | LC |
| *Graphiurus kelleni* | 2015 | LC |
| *Heliosciurus gambianus* | 2008 | LC |
| *Mastomys erythroleucus* | 2008 | LC |
| *Taterillus gracilis* | 2008 | LC |
| *Acomys johannis* | 2008 | LC |
| *Praomys daltoni* | 2008 | LC |
| *Steatomys caurinus* | 2008 | LC |
| *Steatomys cuppedius* | 2008 | LC |
| *Gerbilliscus kempi* | 2008 | LC |
| *Taterillus petteri* | 2008 | LC |
| *Arvicanthis ansorgei* | 2016 | LC |
| *Funisciurus substriatus* | 2008 | DD |
| *Mus mattheyi* | 2016 | LC |
| *Dasymys rufulus* | 2008 | LC |
| *Taterillus pygargus* | 2016 | LC |
| *Lemniscomys zebra* | 2008 | LC |
| *Rattus rattus* | 2014 | LC |
| *Lemniscomys striatus* | 2008 | LC |
| *Gerbilliscus guineae* | 2016 | LC |
| *Arvicanthis rufinus* | 2016 | LC |
| *Hystrix cristata* | 2014 | LC |
| *Praomys derooi* | 2008 | LC |
| *Uranomys ruddi* | 2008 | LC |
| *Fukomys zechi* | 2008 | LC |
| *Hylomyscus pamfi* | 2016 | DD |
| *Mus setulosus* | 2016 | LC |
| *Praomys tullbergi* | 2008 | LC |
| *Funisciurus leucogenys* | 2008 | LC |
| *Dendromus messorius* | 2008 | LC |
| *Graphiurus crassicaudatus* | 2008 | DD |
| *Mus baoulei* | 2016 | LC |
| *Praomys rostratus* | 2016 | LC |
| *Oenomys hypoxanthus* | 2008 | LC |
| *Malacomys edwardsi* | 2008 | LC |
| *Hybomys trivirgatus* | 2008 | LC |
| *Malacomys cansdalei* | 2008 | LC |
| *Heliosciurus punctatus* | 2008 | DD |
| *Anomalurus pelii* | 2009 | DD |
| *Gerbillus nancillus* | 2008 | DD |
| *Gerbillus nanus* | 2016 | LC |
| *Gerbillus tarabuli* | 2016 | LC |
| *Gerbillus gerbillus* | 2008 | LC |
| *Desmodilliscus braueri* | 2008 | LC |
| *Gerbillus henleyi* | 2008 | LC |
| *Jaculus jaculus* | 2008 | LC |
| *Mastomys huberti* | 2016 | LC |
| *Acomys airensis* | 2008 | LC |
| *Taterillus tranieri* | 2008 | LC |
| *Gerbillus rupicola* | 2008 | LC |
| *Taterillus arenarius* | 2008 | LC |
| *Gerbillus pyramidum* | 2016 | LC |
| *Lemniscomys bellieri* | 2008 | LC |
| *Lemniscomys limulus* | 2016 | LC |
| *Gerbilliscus gambiana* | 2008 | LC |
| *Mastomys kollmannspergeri* | 2016 | LC |
| *Gerbilliscus robustus* | 2008 | LC |
| *Taterillus lacustris* | 2008 | LC |
| *Aethomys stannarius* | 2008 | DD |
| *Dasymys foxi* | 2008 | DD |
| *Praomys jacksoni* | 2008 | LC |
| *Dendromus melanotis* | 2016 | LC |
| *Mylomys dybowskii* | 2008 | LC |
| *Epixerus ebii* | 2014 | LC |
| *Dephomys defua* | 2008 | LC |
| *Hybomys planifrons* | 2008 | LC |
| *Oenomys ornatus* | 2008 | LC |
| *Protoxerus aubinnii* | 2008 | DD |
| *Dendromus lachaisei* | 2016 | DD |
| *Grammomys buntingi* | 2008 | DD |
| *Hylomyscus baeri* | 2008 | EN |

**Table B. Mammalian predator species included**

| Academic names | Common names | IUCN | Behavior |
| --- | --- | --- | --- |
| *Vulpes pallida* | Pale fox | LC | Nocturnal |
| *Herpestes ichneumon* | Egyptian mongoose | LC | Diurnal |
| *Herpestes naso* | Long-nosed mongoose | LC | Diurnal |
| *Mellivora capensis* | Honey badger | LC | Nocturnal |
| *Genetta johnstoni* | Johnston's genet | NT | Nocturnal |
| *Nandinia binotata* | African palm civet | LC | Nocturnal |
| *Atilax paludinosus* | Water mongoose | LC | Nocturnal |
| *Crossarchus obscurus* | Cusimanse | LC | Diurnal |
| *Crossarchus platycephalus* | Flat-headed cusimanse | LC | Diurnal |
| *Vulpes zerda* | Fennec fox | LC | Nocturnal |
| *Bdeogale nigripes* | Black-legged mongoose | LC | Nocturnal |
| *Ichneumia albicauda* | White-tailed mongoose | LC | Nocturnal |
| *Mungos mungo* | Banded mongoose | LC | Diurnal |
| *Ictonyx libycus* | Saharan striped polecat | LC | Nocturnal |
| *Ictonyx striatus* | Striped polecat | LC | Nocturnal |
| *Civettictis civetta* | African civet | LC | Nocturnal |
| *Genetta genetta* | Common genet | LC | Nocturnal |
| *Genetta maculata* | Rusty-spotted genet | LC | Nocturnal |
| *Poiana richardsonii* | African linsang | LC | Nocturnal |
| *Genetta servalina* | Servaline genet | LC | Nocturnal |
| *Genetta thierryi* | Haussa genet | LC | Nocturnal |
| *Poiana leightoni* | Leighton's linsang | VU | Nocturnal |
| *Genetta poensis* | King genet | DD | Nocturnal |
| *Genetta pardina* | West african large spotted genet | LC | Nocturnal |
| *Genetta bourloni* | Bourlon's genet | VU | Nocturnal |
| *Felis margarita* | Sand cat | LC | Nocturnal |
| *Felis silvestris* | Wild cat | LC | Nocturnal |
| *Vulpes rueppellii* | Ruppell's fox | LC | Nocturnal |
| *Canis aureus* | Golden jackal | LC | Nocturnal |
| *Canis adustus* | Side-striped jackal | LC | Nocturnal |
| *Leptailurus serval* | Serval | LC | Nocturnal |
| *Caracal caracal* | Caracal | LC | Nocturnal |
| *Genetta cristata* | Crested servaline genet | VU | Crepuscular |
| *Liberiictis kuhni* | Liberian mongoose | VU | Diurnal |
| *Herpestes sanguineus* | Slender mongoose | LC | Diurnal |
| *Lycaon pictus* | African wild dog | EN | Crepuscular |
| *Mungos gambianus* | Gambian mongoose | LC | Diurnal |
| *Caracal aurata* | African golden cat | VU | Nocturnal |

**Table C. Avian predator species included**

| Academic names | Common names | IUCN | Behavior |
| --- | --- | --- | --- |
| *Accipiter ovampensis* | Ovambo sparrowhawk | LC | Diurnal |
| *Buteo auguralis* | Red-necked buzzard | LC | Diurnal |
| *Micronisus gabar* | Garbar goshawk | LC | Diurnal |
| *Melierax metabates* | Dark chanting goshawk | LC | Diurnal |
| *Terathopius ecaudatus* | Bateleur | NT | Diurnal |
| *Aquila rapax* | Tawny eagle | LC | Diurnal |
| *Aquila spilogaster* | African hawk eagle | LC | Diurnal |
| *Lophaetus occipitalis* | Long-crested eagle | LC | Diurnal |
| *Kaupifalco monogrammicus* | Lizard buzzard | LC | Diurnal |
| *Gypohierax angolensis* | Palm-nut vulture | LC | Diurnal |
| *Milvus migrans* | Black kite | LC | Diurnal |
| *Chelictinia riocourii* | Scissor-tailed kite | LC | Diurnal |
| *Hieraaetus ayresii* | Ayres's Hawk-eagle | LC | Diurnal |
| *Circaetus beaudouini* | Beaudouin's Snake-eagle | VU | Diurnal |
| *Hieraaetus wahlbergi* | Wahlberg's Eagle | LC | Diurnal |
| *Accipiter toussenelii* | Red-chested Goshawk | LC | Diurnal |
| *Accipiter badius* | Shikra | LC | Diurnal |
| *Polyboroides typus* | African Harrier-hawk | LC | Diurnal |
| *Accipiter castanilius* | Chestnut-flanked Sparrowhawk | LC | Diurnal |
| *Dryotriorchis spectabilis* | Congo Serpent-eagle | LC | Diurnal |
| *Polemaetus bellicosus* | Martial Eagle | VU | Diurnal |
| *Urotriorchis macrourus* | Long-tailed Hawk | LC | Diurnal |
| *Elanus caeruleus* | Black-winged Kite | LC | Diurnal |
| *Accipiter erythropus* | Red-legged Sparrowhawk | LC | Diurnal |
| *Circaetus cinerascens* | Western Banded Snake-eagle | LC | Diurnal |
| *Circaetus cinereus* | Brown Snake-eagle | LC | Diurnal |
| *Aviceda cuculoides* | African Cuckoo-hawk | LC | Diurnal |
| *Stephanoaetus coronatus* | Crowned Eagle | NT | Diurnal |
| *Macheiramphus alcinus* | Bat Hawk | LC | Diurnal |
| *Aquila africana* | Cassin's Hawk-eagle | LC | Diurnal |
| *Accipiter melanoleucus* | Black Sparrowhawk | LC | Diurnal |
| *Falco alopex* | Fox Kestrel | LC | Diurnal |
| *Falco ruficollis* | Red-necked Falcon | LC | Diurnal |
| *Falco cuvierii* | African Hobby | LC | Diurnal |
| *Falco ardosiaceus* | Grey Kestrel | LC | Diurnal |
| *Falco peregrinus* | Peregrine Falcon | LC | Diurnal |
| *Falco tinnunculus* | Common Kestrel | LC | Diurnal |
| *Falco biarmicus* | Lanner Falcon | LC | Diurnal |
| *Ptilopsis leucotis* | Northern White-faced Owl | LC | Nocturnal |
| *Jubula lettii* | Maned Owl | DD | Nocturnal |
| *Strix woodfordii* | African Wood Owl | LC | Nocturnal |
| *Otus icterorhynchus* | Northern White-faced Owl | LC | Nocturnal |
| *Glaucidium capense* | African Barred Owlet | LC | Nocturnal |
| *Asio capensis* | Marsh Owl | LC | Nocturnal |
| *Otus senegalensis* | African Scops-owl | LC | Nocturnal |
| *Glaucidium perlatum* | Pearl-spotted Owlet | LC | Nocturnal |
| *Bubo poensis* | Fraser's Eagle-owl | LC | Nocturnal |
| *Glaucidium sjostedti* | Sjostedt's Owlet | LC | Nocturnal |
| *Glaucidium tephronotum* | Red-chested Owlet | LC | Nocturnal |
| *Bubo ascalaphus* | Pharaoh Eagle-owl | LC | Nocturnal |
| *Bubo cinerascens* | Greyish Eagle-owl | LC | Nocturnal |
| *Bubo lacteus* | Verreaux's Eagle-owl | LC | Nocturnal |
| *Bubo leucostictus* | Akun Eagle-owl | LC | Nocturnal |
| *Bubo shelleyi* | Shelley's Eagle-owl | NT | Nocturnal |
