# Supplementary material for: An exploration of the protective effect of rodent species richness on the geographical expansion of Lassa fever in West Africa
Source: PLoS Negl Trop Dis. 2021 Feb 1;15(2):e0009108. doi: 10.1371/journal.pntd.0009108 (PMC7877741; doi:10.1371/journal.pntd.0009108)
Supplement: S2 Text — Fig A. Selection process for study area. Note: Made with Natural Earth. (DOCX) [file pntd.0009108.s003.docx]

**S2 Appendix. Selection process for study area**

The selected study area is shown in Fig A. First, we created 1 × 1° grid maps for all African regions. Second, we deleted small grids (< 0.5 × 0.5°) in the marginal area. Third, we excluded grids lacking the reservoir rodent, *Mastomys natalensis*. Fourth, we included only countries with at least one human case of LF before 2017.

**
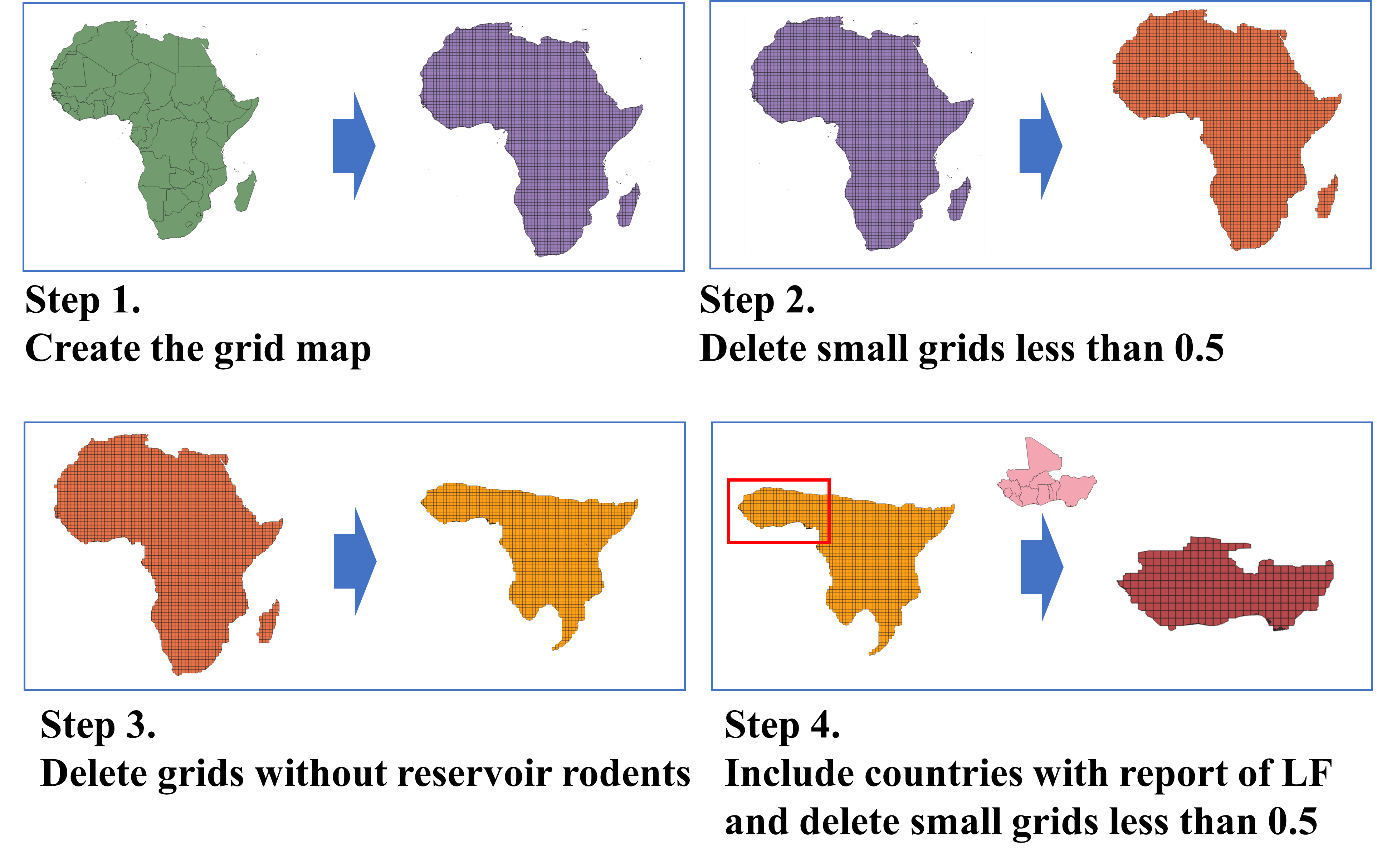
**

**Fig A. Selection process for study area**

Note: Made with Natural Earth.
