# Supplementary material for: An exploration of the protective effect of rodent species richness on the geographical expansion of Lassa fever in West Africa
Source: PLoS Negl Trop Dis. 2021 Feb 1;15(2):e0009108. doi: 10.1371/journal.pntd.0009108 (PMC7877741; doi:10.1371/journal.pntd.0009108)
Supplement: S3 Text — Table A. The list of Lassa fever outbreak regions. (DOCX) [file pntd.0009108.s004.docx]

**S3 Appendix. Historical Lassa fever case report**

Three web-based surveillance datasets were used in this study: ProMed-Mail, HealthMap, and World Health Organization Disease Outbreak News (WHO DON). ProMed-Mail is an expert-led system that includes information on both human and animal outbreaks obtained from the reports of national-level authorities (both official reports and interpersonal communications) and international organizations, and from local news articles. The database is fully open access and has a user-friendly search tool. HealthMap is a database that collects outbreak data from various formal and informal sources; importantly, it provides the geographical coordinates of most cases. The data are not fully accessible on the website, but can be obtained by emailing the HealthMap team. The WHO DON is an official outbreak notification system based on information obtained from local or national public health authorities. Although its sensitivity is similar to that of the above informal data sources, notification regarding new cases is more reliable.

The LF outbreak report data obtained from the web-based surveillance systems and a review were organized into a systematic framework. To maximize sensitivity, we included historical LF cases reported by at least one of the sources. Suspected and human-to-human transmission cases (typically nosocomial) were not included. In terms of imported cases, we included the region of origin if that information was available. The historical LF cases are listed in Table A.

**Table A. The list of Lassa fever outbreak regions**

| **ID** | **Country** | **region** | **Category** | **Year** |
| --- | --- | --- | --- | --- |
| 1 | Benin | Atakora Department | Between 2008 and 2017 | 14, 17 |
| 2 | Benin | Atlantique | Between 2008 and 2017 | 16 |
| 3 | Benin | Alibori | Between 2008 and 2017 | 16 |
| 4 | Benin | Borgou Department | Between 2008 and 2017 | 16, 17 |
| 5 | Benin | Collines | Between 2008 and 2017 | 16 |
| 6 | Benin | Donga Department | Between 2008 and 2017 | 16 |
| 7 | Benin | Oueme Department | Between 2008 and 2017 | 16 |
| 8 | Benin | Plateau | Between 2008 and 2017 | 16 |
| 9 | Ghana | Ashanti | Between 2008 and 2017 | 11 |
| 10 | Ghana | Eastern Region | Between 2008 and 2017 | 11 |
| 11 | Liberia | Bong County | Before 2008 | 90, 07-13, 16 |
| 12 | Liberia | Bomi county | Before 2008 | 90 |
| 13 | Liberia | Grand Bassa | Between 2008 and 2017 | 08-12 |
| 14 | Liberia | Lofa County | Before 2008 | 90, 07-12, 16 |
| 15 | Liberia | Nimba County | Before 2008 | 90, 04, 07, 16-17 |
| 16 | Liberia | Margibi (county) | Between 2008 and 2017 | 14 |
| 17 | Liberia | Montserrado County | Before 2008 | 90, 04 |
| 18 | Mali | Sikasso region | Between 2008 and 2017 | 09 |
| 19 | Mali | Segou region | Before 2008 | 90 |
| 20 | Nigeria | Adamawa State | Before 2008 | 90, 16 |
| 21 | Nigeria | Akwa Iborn State | Between 2008 and 2017 | 15-17 |
| 22 | Nigeria | Anambra State | Between 2008 and 2017 | 08-09, 12, 16-17 |
| 23 | Nigeria | Bauchi State | Between 2008 and 2017 | 14-17 |
| 24 | Nigeria | Benue | Before 2008 | 90, 13 |
| 25 | Nigeria | Borno | Before 2008 | 90, 08 |
| 26 | Nigeria | Cross-River | Between 2008 and 2017 | 17 |
| 27 | Nigeria | Delta State | Between 2008 and 2017 | 17 |
| 28 | Nigeria | East Central State | Before 2008 | 90, 10 |
| 29 | Nigeria | Ebonyi State | Between 2008 and 2017 | 08, 10-17 |
| 30 | Nigeria | Edo State | Before 2008 | 01-05, 08-10, 12-13, 15-17 |
| 31 | Nigeria | Ekiti State | Between 2008 and 2017 | 15-16 |
| 32 | Nigeria | Enugu | Between 2008 and 2017 | 17 |
| 33 | Nigeria | Federal Capital Territory | Between 2008 and 2017 | 09-10, 13, 16 |
| 34 | Nigeria | Gombe State | Between 2008 and 2017 | 12, 15-17 |
| 35 | Nigeria | Imo State | Before 2008 | 90 |
| 36 | Nigeria | Kaduna State | Before 2008 | 90, 15-17 |
| 37 | Nigeria | Katsina | Between 2008 and 2017 | 16 |
| 38 | Nigeria | Kebbi | Between 2008 and 2017 | 10 |
| 39 | Nigeria | Kano State | Between 2008 and 2017 | 15-17 |
| 40 | Nigeria | Kogi State | Between 2008 and 2017 | 15-17 |
| 41 | Nigeria | Kwara | Between 2008 and 2017 | 17 |
| 42 | Nigeria | Lagos State | Between 2008 and 2017 | 08-09, 12, 15-17 |
| 43 | Nigeria | Nasarawa State | Between 2008 and 2017 | 08-09, 12, 14-17 |
| 44 | Nigeria | Ogun State | Between 2008 and 2017 | 08, 15-17 |
| 45 | Nigeria | Ondo State | Between 2008 and 2017 | 09, 12-13, 15-17 |
| 46 | Nigeria | Osun State | Between 2008 and 2017 | 15-16 |
| 47 | Nigeria | Oyo State | Between 2008 and 2017 | 14, 16 |
| 48 | Nigeria | Plateau State | Before 2008 | 90, 92-93, 08, 10, 12-17 |
| 49 | Nigeria | Rivers State | Between 2008 and 2017 | 12-13, 15-17 |
| 50 | Nigeria | Sokoto | Before 2008 | 90 |
| 51 | Nigeria | Taraba | Between 2008 and 2017 | 08, 12-13, 16-17 |
| 52 | Nigeria | Yobe | Between 2008 and 2017 | 12, 16-17 |
| 53 | Sierra Leone | Kailahun District, eastern | Before 2008 | 90, 96-97, 00-01, 03, 16 |
| 54 | Sierra Leone | Tonkolili, northern | Between 2008 and 2017 | 10 |
| 55 | Sierra Leone | Wester Area Rural,western | Between 2008 and 2017 | 14 |
| 56 | Sierra Leone | Bo,southern | Before 2008 | 01, 03, 13 |
| 57 | Sierra Leone | Makeni (village), northern | Between 2008 and 2017 | 10 |
| 58 | Sierra Leone | Kameni (town) | Between 2008 and 2017 | 10 |
| 59 | Sierra Leone | Kenema, eastern | Before 2008 | 90, 95-05 |
| 60 | Sierra Leone | Kono District, eastern | Before 2008 | 01 |
| 61 | Sierra Leone | Pujehun District, southern | Before 2008 | 01 |
| 62 | Sierra Leone | Moyamba District, southern | Before 2008 | 03 |
| 63 | Sierra Leone | Paguma (Eastern) | Before 2008 | 90 |
| 64 | Togo | Oti (district), Savanes | Between 2008 and 2017 | 16-17 |
| 65 | Togo | Kpendjal (district) Savanes | Between 2008 and 2017 | 17 |
| 66 | Burkina Faso | Ouargaye (district) | Between 2008 and 2017 | 17 |
| 67 | Burkina Faso | Como Province | Before 2008 | 90 |
| 68 | Cote d'Ivoire | Beoumi Prefecture | Before 2008 | 90 |
| 69 | Guinea | Faranah Prefecture | Before 2008 | 96-99 |
| 70 | Guinea | Kindia Prefecture | Before 2008 | 96-99 |
| 71 | Guinea | Kissidougou Prefecture | Before 2008 | 96-99 |
| 72 | Guinea | Macenta Prefecture | Before 2008 | 96-99 |
| 73 | Guinea | Nzerekore Prefecture | Before 2008 | 96-99 |
| 74 | Guinea | Pita | Before 2008 | 00 |
| 75 | Guinea | Gueckedou | Before 2008 | 00 |
