# Supplementary material for: An exploration of the protective effect of rodent species richness on the geographical expansion of Lassa fever in West Africa
Source: PLoS Negl Trop Dis. 2021 Feb 1;15(2):e0009108. doi: 10.1371/journal.pntd.0009108 (PMC7877741; doi:10.1371/journal.pntd.0009108)
Supplement: S4 Text — Fig A. Distribution of rodent species richness. Note: The grids (n = 192) were categorized into quintiles of rodent species richness to visualize the geographic distribution. Grey grids (n = 44) indicate regions with incident LF before 2008, which were excluded from the analysis. Made with Natural Earth. Fig B. Distribution of predator species richness. Note: The grids (n = 192) were categorized into quintiles of predator species richness to visualize the geographic distribution. Grey grids (n = 44) indicate regions with incident LF before 2008, which were excluded from the analysis. Made with Natural Earth. Fig C. Distribution of human footprint score. Note: The grids (n = 192) were categorized into quintiles of human footprint score to visualize the geographic distribution. Grey grids (n = 44) indicate regions with incident LF before 2008, which were excluded from the analysis. Made with Natural Earth. Fig D. Distribution of proportion of forest land cover. Note: The grids (n = 192) were categorized into quintiles of proportion of forest land use to visualize the geographic distribution. Grey grids (n = 44) indicate regions with incident LF before 2008, which were excluded from the analysis. Made with Natural Earth. Fig E. Distribution of proportion of agriculture land use. Note: The grids (n = 192) were categorized into quintiles of proportion of agricultural land use to visualize the geographic distribution. Grey grids (n = 44) indicate regions with incident LF before 2008, which were excluded from the analysis. Made with Natural Earth. Fig F. Distribution of elevation. Note: The grids (n = 192) were categorized into quintiles of elevation to visualize the geographic distribution. Grey grids (n = 44) indicate regions with incident LF before 2008, which were excluded from the analysis. Made with Natural Earth. Fig G. Distribution of annual precipitation. Note: The grids (n = 192) were categorized into quintiles of annual precipitation to visualize the geographic [file pntd.0009108.s005.docx]

**S5 Appendix. Geographical distribution of variables included**


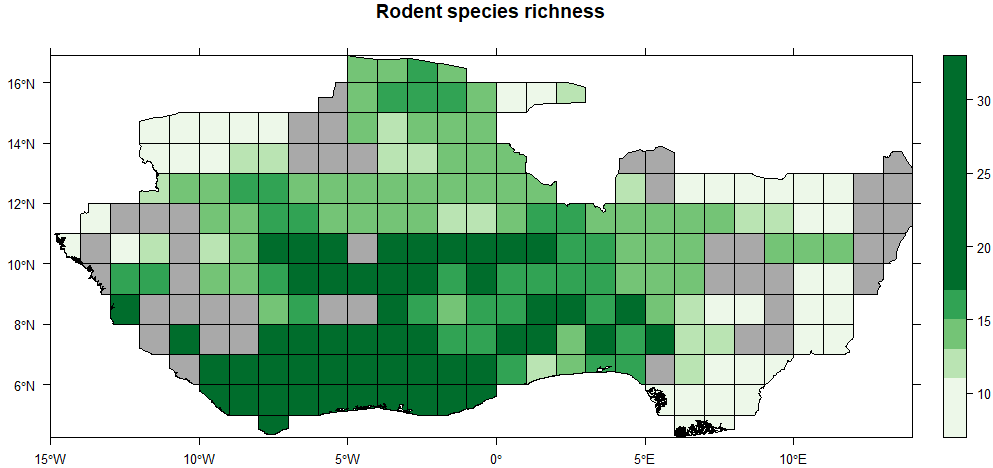


Fig A. Distribution of rodent species richness
*Note*: The grids (n = 192) were categorized into quintiles of rodent species richness to visualize the geographic distribution. Grey grids (n = 44) indicate regions with incident LF before 2008, which were excluded from the analysis. Made with Natural Earth.


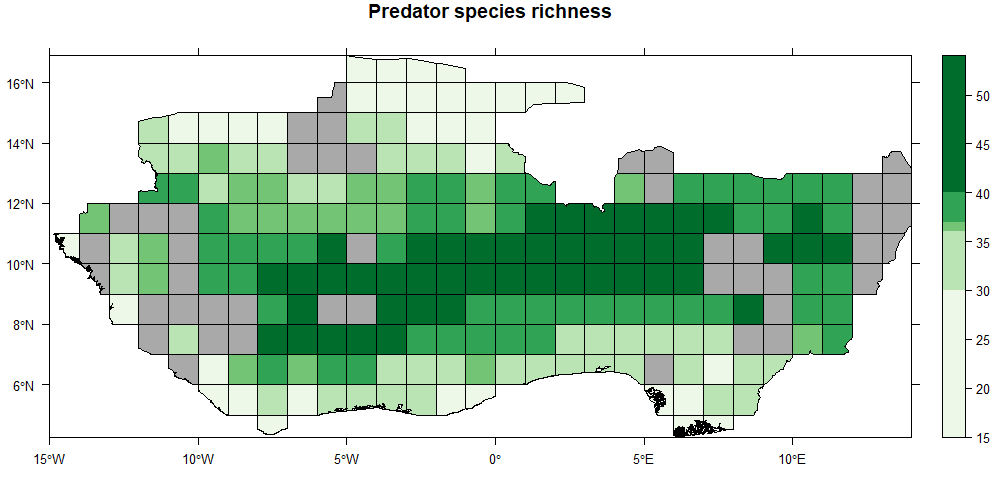


Fig B. Distribution of predator species richness
*Note*: The grids (n = 192) were categorized into quintiles of predator species richness to visualize the geographic distribution. Grey grids (n = 44) indicate regions with incident LF before 2008, which were excluded from the analysis. Made with Natural Earth.


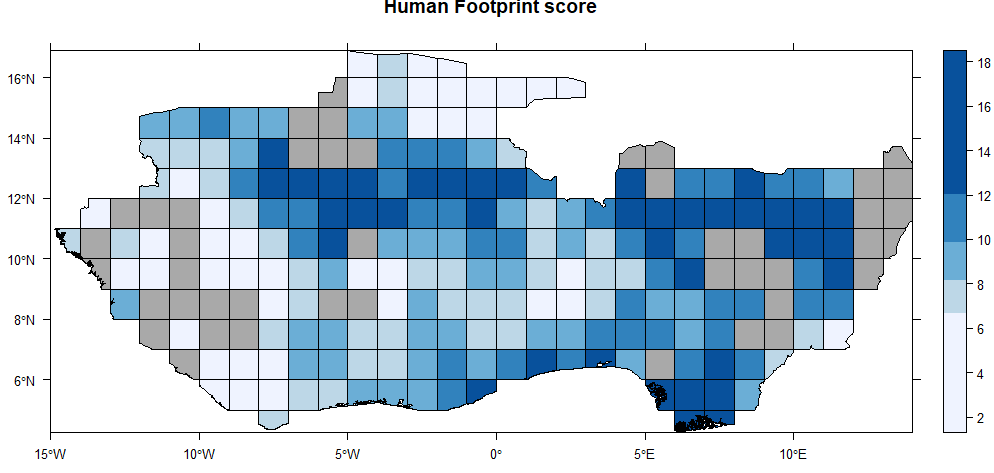


Fig C. Distribution of human footprint score
*Note*: The grids (n = 192) were categorized into quintiles of human footprint score to visualize the geographic distribution. Grey grids (n = 44) indicate regions with incident LF before 2008, which were excluded from the analysis. Made with Natural Earth.


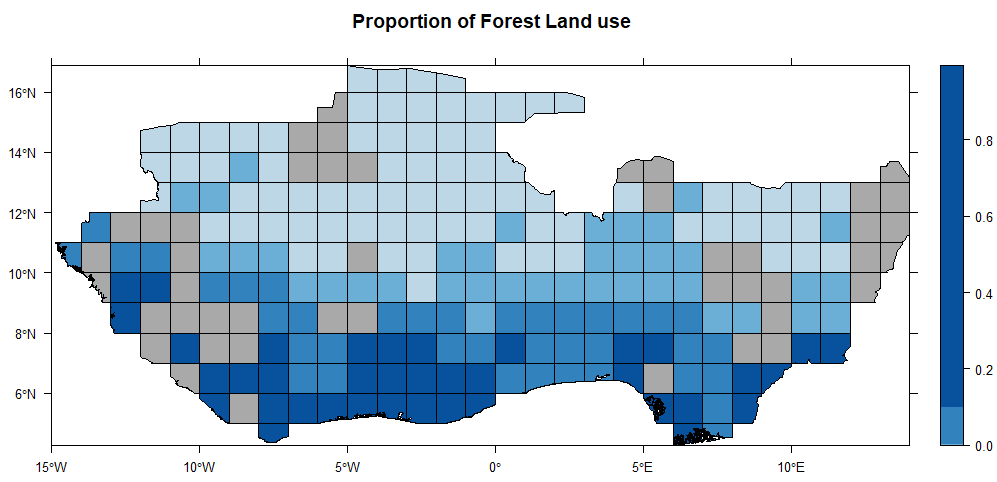


Fig D. Distribution of proportion of forest land cover
*Note*: The grids (n = 192) were categorized into quintiles of proportion of forest land use to visualize the geographic distribution. Grey grids (n = 44) indicate regions with incident LF before 2008, which were excluded from the analysis. Made with Natural Earth.


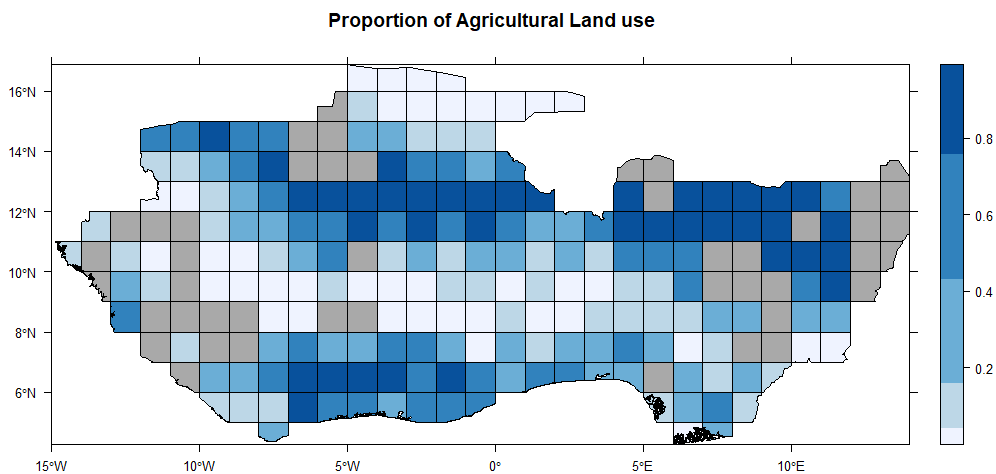


Fig E. Distribution of proportion of agriculture land use
*Note*: The grids (n = 192) were categorized into quintiles of proportion of agricultural land use to visualize the geographic distribution. Grey grids (n = 44) indicate regions with incident LF before 2008, which were excluded from the analysis. Made with Natural Earth.


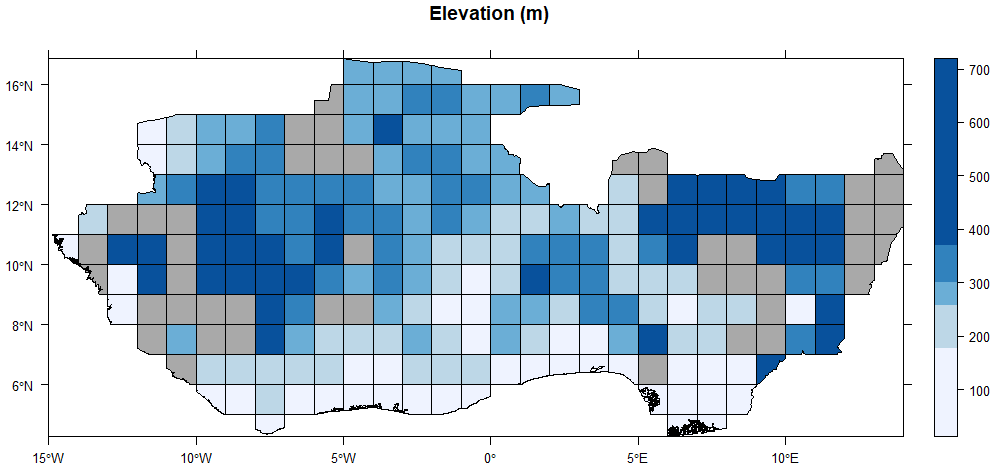


Fig F. Distribution of elevation
*Note*: The grids (n = 192) were categorized into quintiles of elevation to visualize the geographic distribution. Grey grids (n = 44) indicate regions with incident LF before 2008, which were excluded from the analysis. Made with Natural Earth.


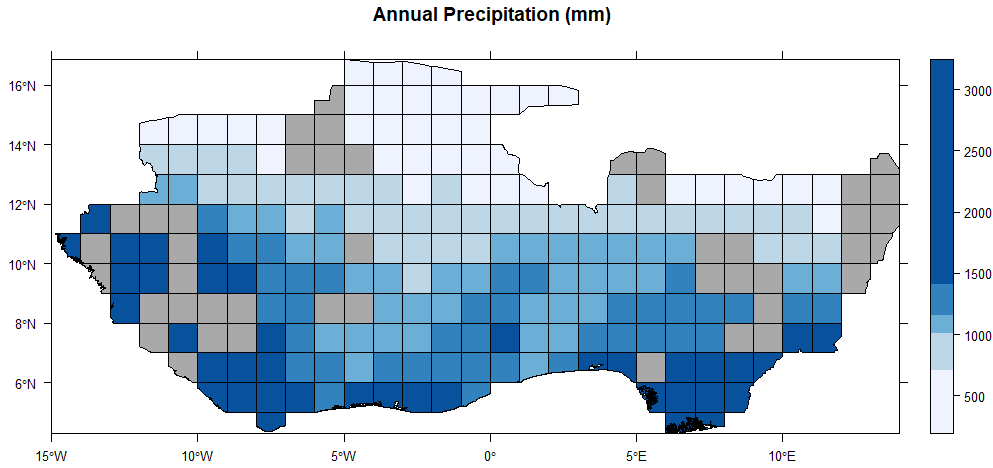


Fig G. Distribution of annual precipitation
*Note*: The grids (n = 192) were categorized into quintiles of annual precipitation to visualize the geographic distribution. Grey grids (n = 44) indicate regions with incident LF before 2008, which were excluded from the analysis. Made with Natural Earth.


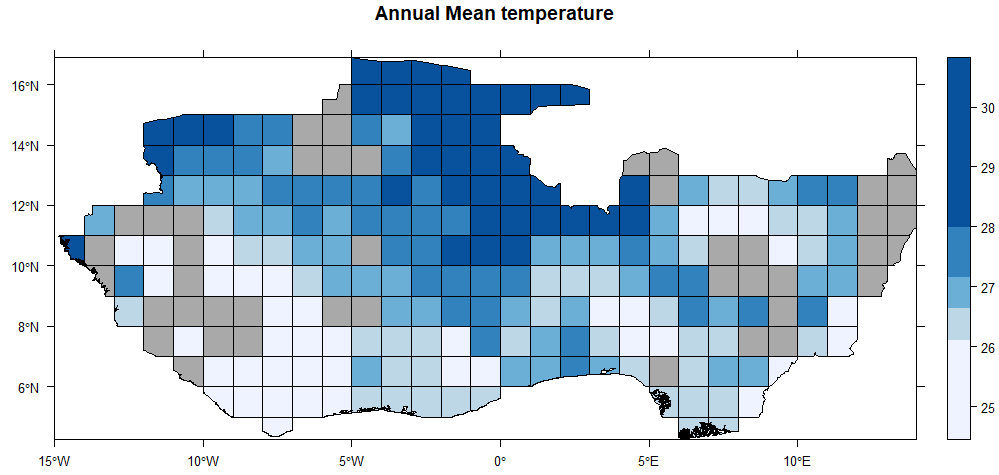


Fig H. Distribution of annual mean temperature
*Note*: The grids (n = 192) were categorized into quintiles of annual mean temperature to visualize the geographic distribution. Grey grids (n = 44) indicate regions with incident LF before 2008, which were excluded from the analysis. Made with Natural Earth.


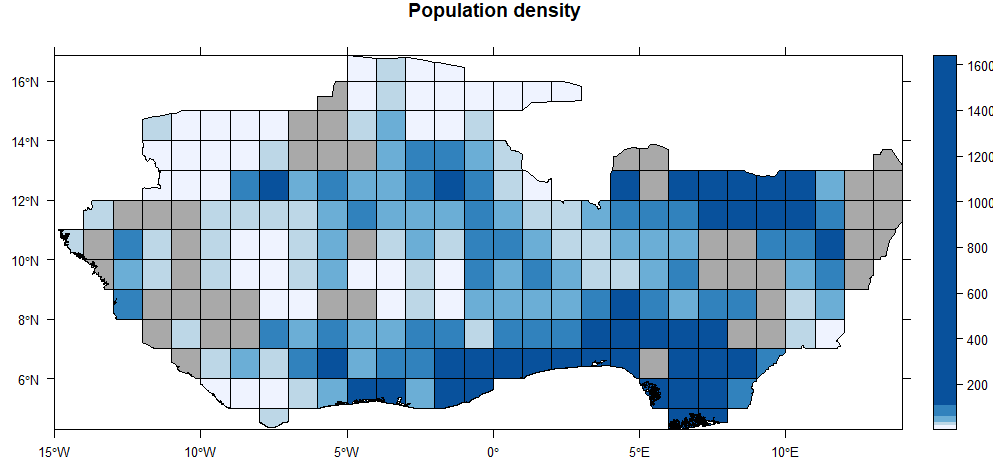


Fig I. Distribution of population density
*Note*: The grids (n = 192) were categorized into quintiles of population density to visualize the geographic distribution. Grey grids (n = 44) indicate regions with incident LF before 2008, which were excluded from the analysis. Made with Natural Earth.


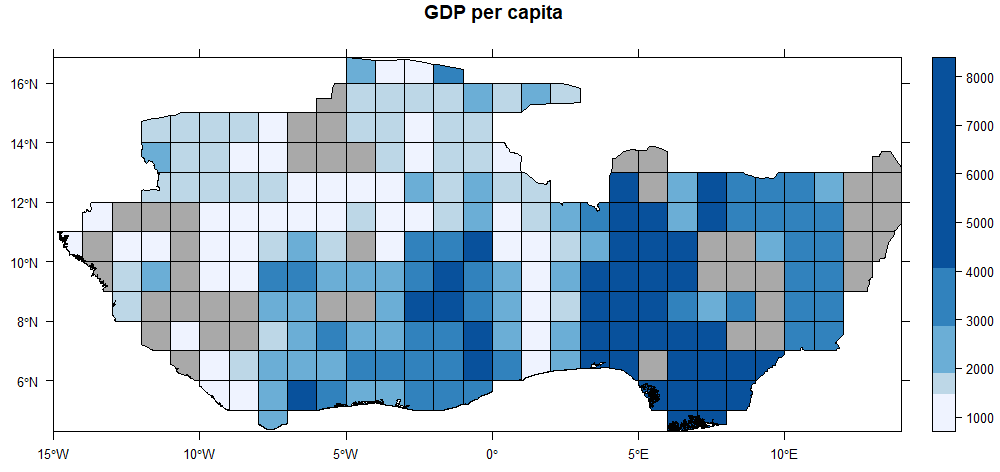


Fig J. Distribution of GDP per capita
*Note*: The grids (n = 192) were categorized into quintiles of GDP per capita to visualize the geographic distribution. Grey grids (n = 44) indicate regions with incident LF before 2008, which were excluded from the analysis. Made with Natural Earth.
