# Supplementary material for: An exploration of the protective effect of rodent species richness on the geographical expansion of Lassa fever in West Africa
Source: PLoS Negl Trop Dis. 2021 Feb 1;15(2):e0009108. doi: 10.1371/journal.pntd.0009108 (PMC7877741; doi:10.1371/journal.pntd.0009108)
Supplement: S5 Text — Table A. Variance inflation factor (VIF) values of the explanatory variables. Fig A. One-to-one correlations of selected variables. Note: A, rodent species richness; B, predator species richness; C, human footprint score; D, proportion of forest land use; E, proportion of agricultural land use; F, elevation; G, total space; H, annual precipitation; I, annual mean temperature; J, population density; K, gross domestic product (GDP) per capita. (DOCX) [file pntd.0009108.s006.docx]

**S6 Appendix. Examination of multicollinearity**

Multicollinearity was examined by calculating the VIF and performing a one‑to-one correlation analysis. Variables with a VIF value of > 10, or that showed a strong correlation with other variables (correlation coefficient > 0.8) were excluded. Because none of the variables met the exclusion criteria, none were excluded.

Table A. Variance inflation factor (VIF) values of the explanatory variables.

| Variables | VIF |
| --- | --- |
| Rodents species richness | 1.70 |
| Predator species richness | 1.86 |
| Human footprint score | 5.03 |
| Forest | 2.70 |
| Cultivated & managed vegetation | 4.23 |
| Elevation | 3.46 |
| Total land space | 1.74 |
| Precipitation | 3.32 |
| Temperature | 4.00 |
| Population density (per km^2^) | 2.62 |
| GDP^1^ (USD per capita) | 1.67 |

^1^GDP, gross domestic product

*Note:* All variables were included in the Poisson model as continuous variables. No variable was excluded (VIF values < 10).


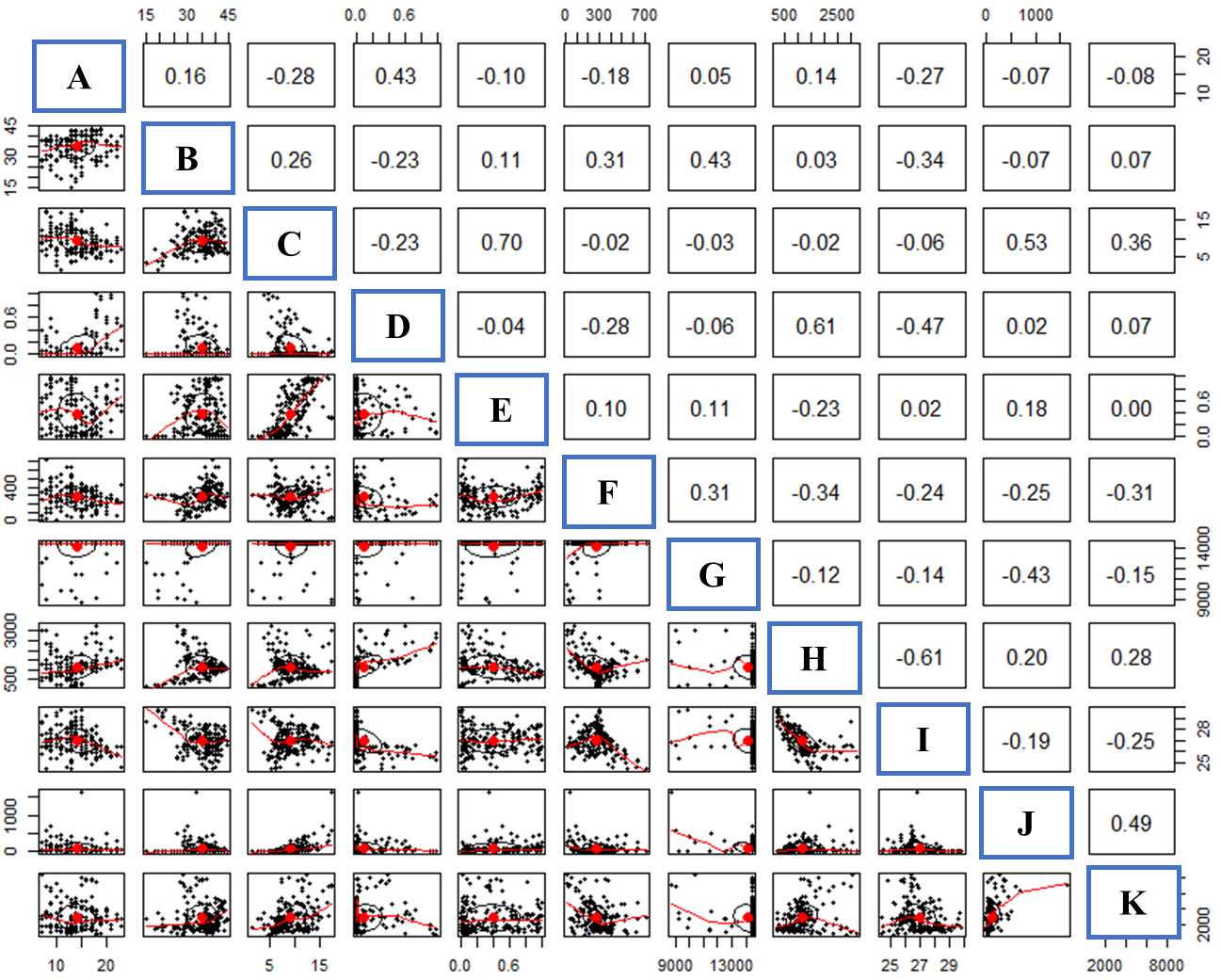


**Fig A. One-to-one correlations of selected variables.**

*Note*: A, rodent species richness; B, predator species richness; C, human footprint score; D, proportion of forest land use; E, proportion of agricultural land use; F, elevation; G, total space; H, annual precipitation; I, annual mean temperature; J, population density; K, gross domestic product (GDP) per capita.
